# Updates in Prostate Cancer Research and Screening in Men at Genetically Higher Risk

**DOI:** 10.1007/s40142-021-00202-5

**Published:** 2021-10-08

**Authors:** Elizabeth K. Bancroft, Holly Ni Raghallaigh, Elizabeth C. Page, Rosalind A. Eeles

**Affiliations:** 1 Urology Genetics, The Royal Marsden NHS Foundation Trust, Downs Road, Sutton, SM2 5PT, UK; 2 Oncogenetics Team, The Institute of Cancer Research, 15 Cotswold Road, Sutton, SM2 5NG, UK

**Keywords:** Prostate cancer, Prostate cancer screening, Family history, Genetic predisposition, Genetic variants, Polygenic risk score

## Abstract

**Purpose of Review:**

Prostate cancer (PrCa) is the most common cancer in men in the western world and is a major source of morbidity and mortality. Currently, general population PrCa screening is not recommended due to the limitations of the prostate-specific antigen (PSA) test. As such, there is increasing interest in identifying and screening higher-risk groups. The only established risk factors for PrCa are age, ethnicity, and having a family history of PrCa. A significant proportion of PrCa cases are caused by genetic factors.

**Recent Findings:**

Several rare germline variants have been identified that moderately increase risk of PrCa, and targeting screening to these men is proving useful at detecting clinically significant disease. The use of a “polygenic risk score” (PRS) that can calculate a man’s personalized risk based on a number of lower-risk, but common genetic variants is the subject of ongoing research. Research efforts are currently focusing on the utility of screening in specific at-risk populations based on ethnicity, such as men of Black Afro-Caribbean descent. Whilst most screening studies have focused on use of PSA testing, the incorporation of additional molecular and genomic biomarkers alongside increasingly sophisticated imaging modalities is being designed to further refine and individualise both the screening and diagnostic pathway. Approximately 10% of men with advanced PrCa have a germline genetic predisposition leading to the opportunity for novel, targeted precision treatments.

**Summary:**

The mainstreaming of genomics into the PrCa screening, diagnostic and treatment pathway will soon become standard practice and this review summarises current knowledge on genetic predisposition to PrCa and screening studies that are using genomics within their algorithms to target screening to higher-risk groups of men. Finally, we evaluate the importance of germline genetics beyond screening and diagnostics, and its role in the identification of lethal PrCa and in the selection of targeted treatments for advanced disease.

## Introduction

Prostate cancer (PrCa) remains one of the major causes of morbidity and mortality among men worldwide with over 1.4 million new cases and 375,000 deaths recorded in 2020 [1–3]. Incidence of PrCa has been increasing in recent decades, and age, ethnicity and having a PrCa family history (FH) are the only established risk factors [4].

The greatest challenge within PrCa screening is developing screening tests that are able to differentiate between indolent, slow-growing tumours and tumours that behave aggressively which require treatment. For men diagnosed with localised low-grade PrCa, the 5-year survival rate is effectively 100%; however, for those with metastatic disease at diagnosis, this reduces to only 30% [4]. The aim is therefore to optimise screening approaches in order to detect clinically significant cancers whilst the disease is treatable to reduce the burden of the disease on men’s lives, whilst at the same time avoiding the harms of overdiagnosis and overtreatment of indolent, screen-detected disease.

### Risk Factors - Age

Incidence of PrCa rises steeply from age 50, with two-thirds of cases being diagnosed over the age of 70 years [2]. However, the incidence of PrCa in men under 55 years is increasing each year. It has been suggested that men with early-onset PrCa can have more aggressive disease and poorer survival than men diagnosed at an older age. These men are likely to be those who carry germline genetic variants that increase their susceptibility to developing PrCa.

### Risk Factors - Ethnicity

PrCa incidence and mortality rates vary across different ethnic groups, with the greatest risk and highest mortality rates seen in men of African ancestry and lowest seen in men of Asian ancestry [5]. In the USA, PrCa incidence in African American men is estimated to be approximately 1.76-fold higher than those of European ancestry and PrCa mortality rates 2.20-fold higher in black men [6]. A large retrospective study in the USA found that whilst overall survival from PrCa is poorer in men of African ancestry compared with men of European ancestry, once they had adjusted for socioeconomic status and matching men by stage, there was no difference in survival. Therefore, this suggests that the majority of the poorer disease outcomes in black men were due to socioeconomic factors. Therefore, genetic variation may play some role, but socioeconomics are likely to play a greater role in Black men [7, 8].

### Risk Factors - Family History

FH is one of the strongest risk factors, with risk increasing with the number of relatives affected and the younger their age at diagnosis [9]. Men with one first-degree relative have an estimated risk of 2.5 times the general population risk. Research in twins has provided evidence for a substantial heritable component in the development of PrCa, estimated to be the most heritable of all common cancers, with 58% heritability [10]. There is evidence for aggressive PrCa clustering within families, including monozygotic twins, suggesting a genetic basis for aggressive disease [11, 12]. It is suggested that familial prostate cancer may be more biologically aggressive than sporadic cancers, with men more likely to relapse and have poorer outcomes after radical prostatectomy [13]. PrCa has also been demonstrated to cluster in families with a strong FH of other cancers, in particular breast cancer [14, 15].

### Risk Factors - Genetic Predisposition

**Fig. 1:**
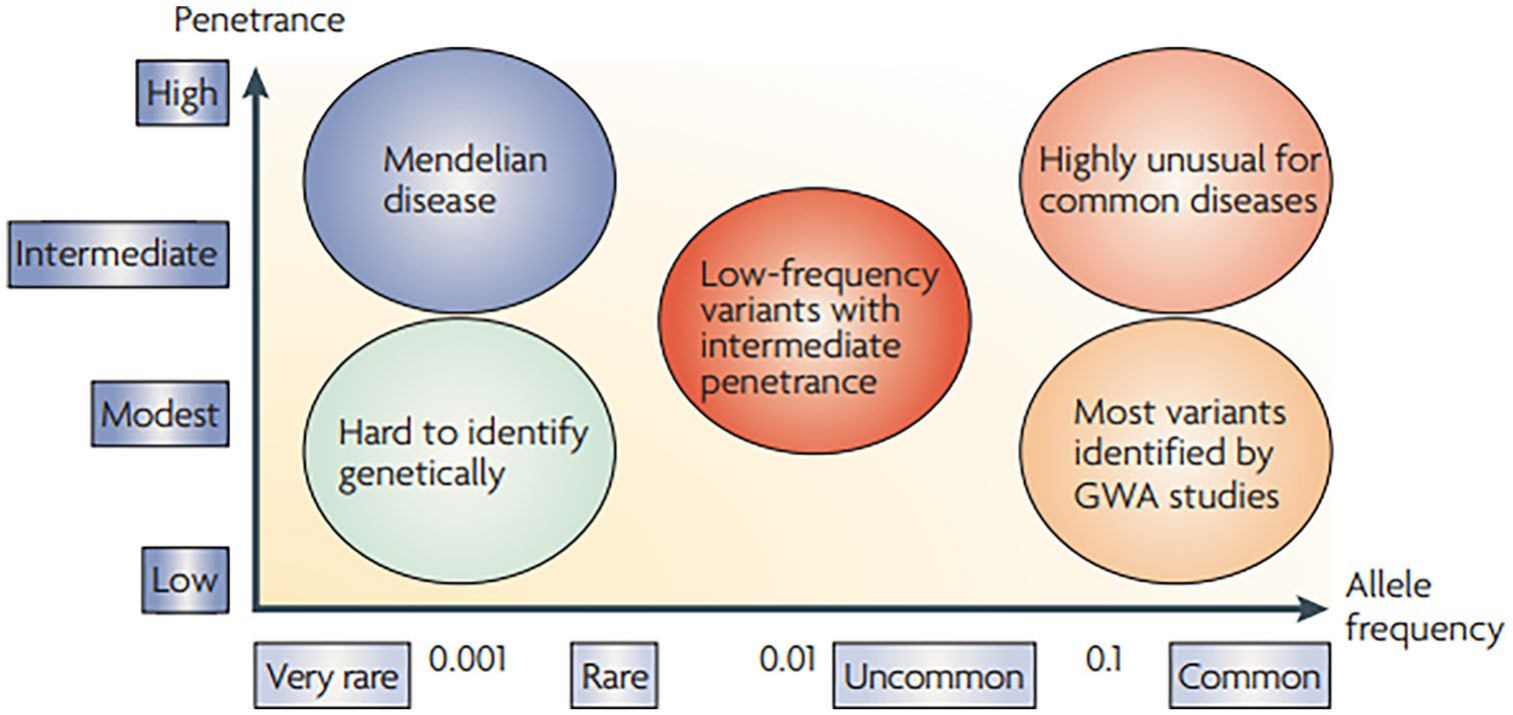
Spectrum of genetic variation in diseases. The X-axis represents the frequency of the variant allele in the general population, the Y-axis represents the penetrance of the variant. Highly penetrant variants responsible for Mendelian disease which occur very rarely in the general population (i.e. Li-Fraumeni Syndrome) fall in the top left of the diagram. Low-penetrance, but common variants such as those discovered for PrCa risk by large-scale GWAS studies are in the bottom right of the graph (red arrow). Reprinted by permission from Springer Nature: Nature Genetics Reviews [21]. Genome-wide association studies for complex traits: consensus, uncertainty and challenges. McCarthy et al. Copyright@2008

There is strong evidence for genetic predisposition to PrCa [16, 17, 18]. There are rare (found in < 1% of men) susceptibility variants, inherited dominantly and that moderately increase the risk of PrCa and common genetic variants (present in > 5% of men) which individually confer a small increased risk of PrCa. Each of these common variants do not individually increase risk to a clinically significant level, but they are thought to act multiplicatively to increase risk to potentially clinically significant levels. Our current knowledge on both rare and common PrCa risk variants is summarised and shown in Fig. 1.

## Prostate Cancer - Genetic Variants

### Dominantly Inherited, Higher Risk Genetic Variants

There is strong evidence that pathogenic variants in DNA repair genes increase the risk of PrCa and predispose to aggressive disease and poorer clinical outcomes [16, 17, 18, 19, 20, 21, 22, 23, 24, 25, 26, 27]. An estimated 8–12% of men with metastatic PrCa have germline mutations in tumour suppressor genes [27, 28, 29]. Men carrying pathogenic variants in the *BRCA2* gene aged < 65 years old have an estimated relative risk of PrCa of 2.5–8.6-fold [30, 31, 32] and a significantly increased risk of early-onset, aggressive disease [20, 21, 22, 26, 33, 34, 35, 36]. Other DNA repair genes have been implicated as conferring a moderate-PrCa risk in studies of men with young onset or metastatic PrCa and require further investigation in larger prospective cohorts. These include *BRCA1* (estimated 1.8–3.5-fold increased risk) [37, 38], *ATM* (odds ratio of 4.4) [20, 39], *CHEK2* (odds ratio of 1.8–8.2) [40, 41], *PALB2* (odds ratio of 3.5) [40, 42] and *HOXB13 *G84E variant (odds ratio of 0.95–93) [40, 43, 44, 45]. The ranges of these risk estimates are wide as pathogenic variants in these genes are rare, and the data have been ascertained from different cohorts of men; for example, case–control studies selected for family history, or unselected cases with aggressive disease. For *CHEK2*, most of the data relate to the 1100delC variant, but other pathogenic variants in *CHEK2* have also been implicated in increasing risk of aggressive disease. For *HOXB13*, risk estimates relate to the missense mutation G84E, which is a founder mutation in Nordic populations, with carrier frequencies reported to be 0.2–1.4% and 0.1–0.5% in other Western European populations.

The DNA mismatch repair genes *MSH2*, *MSH6* and *MLH1* are reported to increase PrCa risk between 2- and tenfold [46, 47, 48, 49, 50] with a meta-analysis by Ryan et al. reporting a 2.13-fold increased risk of PrCa [50]. There is also evidence to suggest an association with higher-grade tumours and younger age of onset [47, 48, 51] associated with pathogenic variants in *MSH2* compared to the other MMR genes [46, 48, 49, 52, 53, 54]. Other genes such as *RAD51D*, *TP53*, *BRIP1* and *NBN* [27, 55, 56] have also been proposed as potentially involved in PrCa development. Research is required to further clarify the level of risk conferred by pathogenic variants in each of these genes and the association of these variants with aggressive disease and age at onset to inform their inclusion in gene panel tests [9, 57].

There is debate surrounding the role of germline genetic testing for PrCa, whom should be offered it and when. Germline testing in men with PrCa is primarily recommended to inform treatment options or clinical trial eligibility for metastatic or locally advanced disease. The National Comprehensive Cancer Network (NCCN) Clinical Practice Guidelines in Oncology: Prostate Cancer (2021) recommend panel testing alongside genetic counselling for men with high-risk localised, regional or metastatic disease, Ashkenazi-Jewish ancestry, a FH of high-risk germline pathogenic variants; a strong FH of breast cancer, ovarian cancer or PrCa with a gene panel test that includes *ATM*, *BRCA1*, *BRCA2*, *MSH2*, *MLH1*, *MSH6*, *PMS2*, *HOXB13*, *PALB2* and *CHEK2* [58﻿]. In the UK, the NHS Genetic Test Directory (https://www.england.nhs.uk/publication/national-genomic-test-directories) is not currently written with routine testing of PrCa patients in mind, being restricted to men with a strong family history of breast and ovarian cancers where the risk of detecting a pathogenic variant is > 10% using the CanRisk tool (https://ccge.medschl.cam.ac.uk/canrisk), or with a Manchester Score > 15 [59]. The test directory is regularly reviewed, and we anticipate that future versions will contain standard testing pathways for men with PrCa where there is a strong likelihood of detecting a pathogenic variant.

In unaffected men, germline testing is increasingly advocated within the context of a FH, or for patients with Ashkenazi Jewish ancestry [57]. The NCCN Prostate Cancer Early Detection [60] and Genetic/Familial High-Risk assessment [61] guidelines recommends referral to a genetics professional if there is a known, or suspected cancer susceptibility pathogenic gene variant. If an unaffected man has a personal history of male breast cancer or exocrine pancreatic cancer, Ashkenazi-Jewish ancestry, a probability of > 5% of carrying a *BRCA2* pathogenic variant based on a probability model, having a first or second-degree relative with any of the following: breast cancer aged ≤ 45, breast cancer aged 46–50 with a second breast cancer diagnosed at any age, breast cancer aged 46–50 with ≥ close blood relative with breast, pancreatic, ovarian or prostate cancer [61].

### Common Genetic Variants and Polygenic Risk Score

Common genetic variants, or single nucleotide polymorphisms (SNPs), occur in > 5% of the population. Large-scale genome-wide association studies (GWAS) have led to the discovery of over 200 SNPs associated with PrCa risk (reviewed in [9]). Each SNP has a low penetrance and confers a small increase in PrCa risk if occurring alone, but are thought to act multiplicatively. Over 30% of the familial risk in PrCa can be explained by the SNPs identified to date, with men in the top 1% of the risk profile having a 5.7-fold increase in risk of developing PrCa compared with the average population risk [62]. Therefore, a number of men in the upper end of the risk distribution may have an elevated risk similar to that of men who carry mutations in moderate-risk genes [26]. These advances in our knowledge about common variants and their contribution to risk of PrCa are therefore ready for implementation in clinical studies evaluating their use in population risk-stratification [63]. A polygenic risk score (PRS) can be calculated from a man’s PrCa risk-SNP genotype to estimate his individual risk of developing PrCa relative to the average population risk. This genotyping is a much cheaper option than the cost of the sequencing of gene panels, to screen for rare pathogenic mutations. There is also evidence to suggest that stratifying screening to men at the highest-risk of PrCa, based on their PRS, could potentially reduce overdiagnosis and improve the cost-effectiveness of a PrCa screening programme [64].

Currently, most GWAS data are from European populations, and so, the calculation of PRS in men of other ethnicities requires further consideration. Multi-ethnic GWAS have found that many (but not all) PrCa risk SNPs are shared between populations; however, the risk associated with a variant may vary according to ethnicity. Recently, Darst et al. reported the importance of a SNP specific to men of African ancestry and its role in increasing these men’s risk of PrCa at an early age [42]. A recent multi-ethnic GWAS has identified 86 new SNPs associated with increased PrCa risk in men of African ancestry, and of interest, these are associated with higher-per-allele odds ratios than many of those in European men [65].

The modifying effect of SNPs in men with known pathogenic variants in cancer predisposition genes is an area of increasing interest. Lecarpentier et al. (2017) genotyped approx. 1800 men with known *BRCA1* or *BRCA2* variants for 103 known PrCa risk SNPs [66]. Large differences in absolute cancer risks were seen at the extremes of the PRS distribution in their *BRCA* cohort. For example, PrCa risk at age 80 at the 5th and 95th percentiles of the PRS varied from 7 to 26% for carriers of *BRCA1* mutations and from 19 to 61% for carriers of *BRCA2* mutations, highlighting that a PRS can further inform us of heritable risk in such men and help further risk stratify this already high-risk cohort.

### Genetic Predisposition to Prostate Cancer - Summary

PrCa risk is influenced by a combination of common and rare germline variants, with rare variants important within specific families and sub-groups and common variants a substantial contributor at the population level [9]. Identifying an underlying genetic predisposition to PrCa is important for a number of reasons. For those identified at higher risk, tailored or targeted screening protocols can be implemented. For men receiving a diagnosis of locally advanced or metastatic disease, there are treatment implications relating to the use of targeted, molecular therapy if a germline variant is present. Finally, there is the critical opportunity for cascade testing amongst family members in those with moderate/higher risk variants. Cascade testing is important as many of the moderate risk genes implicated in the development of PrCa are well-established to predispose to several cancers, for which screening and risk-reducing measures may be available. Pathogenic variants in genes such as *BRCA1*, *BRCA2* and MMR genes are well characterised with clear clinical management guidelines. There are also family planning options such as pre-implantation genetic diagnosis that can be explored.

## Prostate Cancer Screening

The prostate-specific antigen (PSA) test is the most commonly utilised PrCa biomarker currently available but is not recommended as a general population screening tool due to its well-documented limitations [67, 68]. Data from large screening trials including the European Randomised Study of Screening for Prostate Cancer (ERSPC) [69, 70] and Prostate, Lung, Colorectal and Ovary screening study (PLCO) [71, 72] do not support population screening using PSA, despite evidence of a 21% reduction in PrCa-specific mortality after 13 years of follow-up, and evidence to support earlier diagnosis increasing the likelihood of cure [70]. The major limitation is the inability of PSA to discriminate between clinically significant cancer and indolent disease that will not affect a man during his lifetime. The harms of ‘unnecessary’ prostate biopsies resulting in overdiagnosis and over treatment of indolent cancers, together with the psychological burden of life-changing side effects of treatments such as incontinence and erectile dysfunction, are felt to outweigh any benefit in potential mortality reduction. It is not feasible to use expensive imaging techniques, for example MRI-fusion technologies, at a population level without limiting numbers by classifying men into different risk categories. Although there has been much focus on new molecular and genomic biomarkers for diagnosing PrCa [73], to date, prostate-specific antigen (PSA) remains the only biomarker used as a screening tool clinically in routine practice. There is therefore an urgent need for new biomarkers to be used alongside, or in place of, PSA to optimise the diagnostic pathway, ensuring only men who are likely to benefit from treatment are screened, i.e. screening tests with the ability to identify men at highest risk of developing clinically significant and potentially life-limiting tumours.

As described above, certain groups of men have a higher risk of early onset and aggressive PrCa. Table 1 summarises the current international PrCa screening recommendations for higher risk groups. Most screening advisory bodies recommend PSA screening for men with a FH of PrCa and men of African ancestry, with the EAU recently updating their screening guidelines to include yearly PSA screening in men with pathogenic germline *BRCA2* variants from age 40 [74]. The NCCN guidelines advise PrCa screening to start at age 45 for male *BRCA2* carriers and to consider the same for *BRCA1* carriers [60]. There are a number of screening studies in progress, which are stratifying men into different risk groups based on different risk factors and aiming to use this risk-stratification, to improve on using PSA screening alone. These studies are summarised as follows. We predict that screening guidelines will be expanded to include men with other germline variants over the coming years based on the results of these studies. The benefit of risk-stratifying men based on genetic markers is that they are stable throughout a man’s lifetime and not influenced by disease processes.

**Table 1:** Summary of specific screening advice using PSA in specific high-risk groups and general population

	**General population screening**	**Screening in men with a family history**	**Screening in Black men**	**Screening in *BRCA2* carriers**
American Urological Association^1^	No	Yes from 40 years*	Yes from 40 years	Not specified
American Cancer Society^2^	No	Yes from 40–45 years	Yes from 40–45 years	Not specified
European Association of Urology^3^	No	Yes from 45 years	Yes from 45 years	Yes from 40 years
National Comprehensive Cancer Network (NCCN, USA)^4^	To consider in all men aged 45–75	Yes from 45 years	Yes from 45 years	Yes from 45 years
National Institute of Health and Care Excellence (UK)^5^	No	Not specified	Not specified	Not specified
US Preventive Services Taskforce^6^	No	Not specified	Not specified	Not specified

^1^ https://www.auanet.org/guidelines/guidelines/prostate-cancer-early-detection-guideline#x2638

^2^ https://www.cancer.org/cancer/prostate-cancer/detection-diagnosis-staging/acs-recommendations.html

^3^ https://uroweb.org/guideline/prostate-cancer/

^4^ https://www.nccn.org/guidelines/guidelines-detail?category=2&id=1460

^5^ https://www.nice.org.uk/guidance/ng131

^6^ https://www.uspreventiveservicestaskforce.org/uspstf/recommendation/prostate-cancer-screening

## Screening in Carriers of Rare Variants

### *BRCA1* and *BRCA2*

The IMPACT study (https://clinicaltrials.gov/ct2/show/NCT00261456) has been underway since 2014 to assess the utility of targeted PSA screening for early diagnosis in germline *BRCA1* and *BRCA2* pathogenic variant carriers [34, 75]. A total of 2932 men (919 *BRCA1* and 909 *BRCA2* pathogenic variant carriers) aged 40–69 were enrolled for annual PSA screening, with prostate biopsy indicated where PSA is > 3.0 ng/ml. IMPACT was the first prospective study to use germline genetic markers for identifying men with a high risk of PrCa. After 3 years of screening, men with a pathogenic variant in *BRCA2* were found to have a higher incidence of PrCa per 1000 person years (19.4 vs 12.0; *p* = 0.03), were younger at diagnosis (61 vs 64 years; *p* = 0.04) and had more clinically significant disease (77% vs 40%; *p* = 0.01) compared with non-carriers. Therefore, the results of IMPACT so far confirm that PSA screening achieves early detection of aggressive PrCa in *BRCA2* carriers. The results of the full 5 years of screening are required to confirm the role of screening in men with pathogenic variants in *BRCA1* and to help develop the optimal screening strategy. Segal et al., in 2020, reported their first round of PrCa screening in *BRCA1* and *BRCA2* carriers, using an approach combining age-stratified PSA and MRI. They found a cancer detection rate of 8.6%, with a significant benefit of screening using MRI compared to PSA in young men aged 40–55, whereas PSA had the highest benefit in those aged > 55 [76].

### Mismatch Repair Genes

The IMPACT study was extended to include a cohort of men with MMR genes *MSH2*, *MSH6* and *MLH1* using the same screening algorithm as above. The cohort of 828 participants has completed recruitment and includes 204 *MLH1* carriers, 305 *MSH2* carriers and 135 *MSH6* carriers and 586 controls (men who had tested negative for a known familial pathogenic variant). Annual PSA screening will continue until 2024, and the baseline results are to be due to be submitted for publication imminently (Bancroft et al., 2021; personal communication). This will be the first published prospective screening study in men with pathogenic variants in these MMR genes.

#### Screening Using Common Variants

Using modelling, there is evidence to suggest that incorporating common variants into risk-stratification, and screening models could improve PrCa detection. Xu et al. (2009) built a risk-prediction model using 14 known PrCa-associated SNPs together with FH [77]. They found an odds ratio of 4.92 for developing PrCa for men with a positive FH and with ≥ 14 risk alleles [77]. Zheng et al. evaluated the effect of five SNPs associated with PrCa in a risk model combining FH and found their model accounted for 46% of PrCa cases within their cohort, with an odds ratio of 9.46 compared with men with no risk factors; this risk was independent of PSA [78].

The PROFILE study (https://clinicaltrials.gov/ct2/show/NCT02543905) is in progress, examining the role of upfront MRI and prostate biopsy (regardless of PSA) in men aged 40–69 from two high-risk groups: 350 men with a FH of PrCa and 350 men of Afro-Caribbean descent. The study aims to calculate each participant’s PRS and determine the PRS score association with MRI/biopsy outcome and its utility in detecting clinically significant PrCa. Men declining MRI and biopsy will undergo yearly PSA screening for a minimum of 5 years.

The PROFILE pilot study evaluated 115 men of European ancestry with a FH to establish feasibility and acceptability of the protocol. One hundred men underwent prostate biopsy at study enrolment, and the results were correlated with clinical variables and PRS based on 71 SNPs. The study reported a cancer detection rate of 25%, of which 48% were clinically significant cancers requiring radical treatment [79]. No association was detected between the PRS and biopsy outcome; however, this initial pilot study was not powered to detect this difference. The main study is using a more extensive PRS with > 150 SNPs and will provide valuable data on the ability of the PRS to predict clinically significant disease and determine its utility in the screening of higher risk men.

#### Stockholm 3

The Stockholm 3 (STHLM3) study, a Swedish prospective screening study involving 58,818 participants, aged 50 to 69 years, compared two cohorts of men, one undergoing PSA only versus a cohort of men undergoing the STHLM3 model [80]. The STHLM3 model predicts the probability of clinically significant PrCa based on a combination of plasma protein biomarkers (PSA, free PSA, intact PSA, hK2, MSMB, MIC1), genetic variants (232 SNPs) and clinical variables (age, FH, previous prostate biopsy, prostate examination). They demonstrated that compared with offering biopsy to all men with a PSA > 3.0 ng/mL, the STHLM3 model decreased overdiagnosis by avoiding 32% of prostate biopsies without significantly decreasing sensitivity to detect high-grade disease (Gleason ≥ 7) and reducing the number of low-grade cancers (Gleason ≤ 6) detected by 17% [80]. The model has been shown to be cost-effective compared with a screening programme based on PSA alone [81]. However, all biomarkers in the model were added in one step and so it is not possible to analyse the individual effect of each biomarker and understand the proportional contribution of each factor [82]. The effect of STHLM3 plus MRI has also been studied, demonstrating that by biopsying men with a positive STHLM3 and a positive MRI, 38% of biopsies could have been avoided, and this would have missed only 8% of all clinically significant PrCa compared with using MRI alone [83]. The STHLM3-MRI study is underway to determine whether use of MRI-fusion within the study algorithm can further refine and improve the diagnostic pathway [84].

#### BARCODE1 Study

The BARCODE1 study (https://www.clinicaltrials.gov/ct2/show/NCT03857477) is the first prospective study to assess utility of PRS genetic profiling in a general practice setting for risk stratification. The study is enrolling men of European ancestry from General Practitioners (GPs) in the UK, to provide a DNA sample, from which the PRS is calculated using 130 PrCa risk SNPs. Men whose PRS puts them in the top 10% of the genetic risk distribution are invited to undergo screening with MRI and prostate biopsy, and those with negative screening tests continue annual PSA for 5 years. The study aims to recruit a total of 5000 men.

## Impact of Genetic Status on Treatments and Outcomes

Most men with PrCa present with localised and treatable disease; however, the classification of tumour characteristics into low, intermediate or high-risk of metastasis at diagnosis is important for informing treatment strategy [85]. Indolent cancers are usually treated with active surveillance, whereas those with aggressive features warrant more intensive treatments such as surgery, radiotherapy or focal therapies ± adjuvant therapies with hormones and chemotherapy. From a treatment perspective, knowing germline pathogenic variant status is increasingly important, particularly within the metastatic PrCa context. There is potential for the use of genetic testing at diagnosis to alter the treatment pathway in men with localised disease with data to suggest that for men on active surveillance, there is a higher upgrading on rebiopsy in men with germline pathogenic variants in *ATM*, *BRCA1* or *BRCA2* compared with non-carriers [20]. Therefore, men undergoing active surveillance identified with a high-risk pathogenic variant would be preferentially offered radical treatment. Approximately, one-third of men receiving treatment such as surgery or radiotherapy with curative intent will experience a recurrence of their cancer [86]. Germline pathogenic variants in DNA repair genes, *BRCA1*, *BRCA2* and *ATM*, have been associated with aggressive behaviour of localised PrCa, cancer-specific mortality and death from PrCa at a younger age [22, 25, 31, 87]. Therefore, when considering treatment options, the presence of germline pathogenic variants should be taken into account and should steer clinicians away from opting for active surveillance in favour of surgery in men with PrCa and a pathogenic germline variant [20, 36].

Castro et al. investigated the tumour characteristics and treatment outcomes in a cohort of 1302 men with PrCa, including 67 men with germline *BRCA1* and *BRCA2* pathogenic variants. They found that *BRCA1* and *BRCA2* pathogenic variant carriers had more aggressive tumours, higher T scores, higher Gleason scores and more frequent nodal involvement, developing metastasis sooner and having shorter overall and cause-specific survival at 10 years compared with non-carriers. Of those treated with curative intent, treated with either radical prostatectomy or radiotherapy, there was no significant difference detected in PrCa-specific survival between *BRCA1* and *BRCA2* carriers and non-carriers who underwent prostatectomy; conversely, there was a significant difference in PrCa-specific survival detected between *BRCA1* and *BRCA2* carriers and non-carriers who underwent radiotherapy [21, 22]. These retrospective studies had a relatively small number of *BRCA1* and *BRCA2* carriers, and further evaluation within the GENPROS study (https://clinicaltrials.gov/ct2/show/NCT02705846) aims to assess the clinical outcomes in men with PrCa and germline pathogenic variants in PrCa predisposition genes, including the *BRCA1*, *BRCA2*, *ATM*, *CHEK2*, *PALB2*, *HOXB13* and MMR genes (*MSH2*, *MSH6*, *MLH1*, *PMS2*) amongst others.

### PARP Inhibitors

The PARP-inhibitor Olaparib is approved for the treatment of advanced ovarian and breast cancers associated with germline *BRCA1* or *BRCA2* pathogenic variants [88]. The use of olaparib in men with metastatic castration-resistant PrCa (mCRPC) with germline or somatic pathogenic variants in *BRCA1* or *BRCA2* has been evaluated in the TOPARP studies [24, 89]. These studies confirmed that PARP inhibitors are associated with an increased response rate in this sub-group of men. TOPARP-B also identified a potential role for PARP inhibitors for men with metastatic disease with somatic or germline pathogenic variants in *ATM*, *PALB2*, *FANCA* or *CHEK2*, although further data are needed to precisely assess the clinical benefit for each gene. PARP inhibitors are now licensed in the USA and Europe for men with germline pathogenic variants in DNA repair genes, specifically *BRCA1*, *BRCA2* and *ATM* [39, 89, 90].

### Immune Checkpoint Inhibitors

Men with prostate tumours that are MMR deficient are known to be sensitive to immune checkpoint inhibitors [91, 92]. The Philadelphia Prostate Cancer Consensus (2017) recommended that men with PrCa and a FH of Lynch Syndrome should be screened for MMR gene variants, and men whose prostate tumour contains pathogenic variants in MMR genes should undergo germline testing [93]. The NCCN guidelines support the use of pembrolizumab in patients with MMR-deficient metastatic castrate-resistant PrCa whose disease has progressed on at least one line of treatment [94, 95].

### Platinum Chemotherapy

Men with PrCa and pathogenic variants in *BRCA1*, *BRCA2* and other DNA repair genes have also shown an increased sensitivity to platinum chemotherapy [96–98]. *BRCA1*/*BRCA2* pathogenic variant status is known to predict response to platinum-based chemotherapies in other cancers, predominantly breast and ovarian cancer, but they are not routinely used to treat PrCa due to a lack of proven clinical benefit in unselected populations [99]. There are data to support a similar response in men with mCRPC who have a germline variant in a DNA repair gene and especially in patients with mCRPC and *BRCA2* pathogenic variants [97, 98]. The BARCODE2 study (https://clinicaltrials.gov/ct2/show/NCT02955082) is aiming to investigate response to carboplatin in men with mCRPC who have completed all lines of standard treatment and who harbour germline pathogenic variants in a DNA repair gene, some of which have not previously been assessed in the above trials. Platinum chemotherapy is an attractive prospect as it has the advantage of being readily and cheaply available within Oncology.

## Conclusions

The importance of understanding germline genetic variation and its role in identifying men at increased risk of PrCa for targeted screening and informing targeted treatment decisions in men with advanced disease is becoming increasingly recognised [9]. A combination of common and rare variants is likely to influence risk of PrCa, with common variants conferring a substantial contribution at the general population level and rare variants specific to certain families or populations.

Studies are showing the promise of PRS within a PrCa screening algorithm for risk-stratification and facilitation of early detection of PrCa in men at higher risk. Both men with a FH and those of African ancestry have a susceptibility to earlier onset and more aggressive disease making them ideal cohorts to establish robust screening protocols to improve early diagnosis and treatment. However, whilst studies assessing the feasibility of using genetics for targeted screening exist, further research is required, particularly in respect to our understanding of the contribution of common variants across diverse ethnic groups, to be able to offer genetic risk assessments at a general population level [9]. Additionally, the contribution of common variants towards risk of aggressive disease individually or cumulatively requires further research.

There is growing demand from patients for routine integration of genetics into oncological care. Whilst such routine integration has been achieved within ovarian and breast cancer, there is huge potential for a similar model to be implemented in PrCa. We anticipate that the routine use of genetic testing, incorporating both common and rare variants, will be integrated into PrCa screening and management protocols within the next 5 years internationally. This integration will have a direct impact on PrCa screening accuracy and efficiency, early diagnosis and treatment options, and ultimately will improve PrCa survival in the highest-risk populations of men, together with inevitable health economic and psychological benefits [7•, 20•, 23•, 26, 27, 28, 34•, 42•, 49, 50, 51, 52•, 57•, 61, 64•, 69•, 76••, 83•].
